# Predicting sepsis treatment decisions in the paediatric emergency department using machine learning: the AiSEPTRON study

**DOI:** 10.1136/bmjpo-2024-003273

**Published:** 2025-05-14

**Authors:** Sylvester Gomes, Harpreet Dhanoa, Phil Assheton, Ewan Carr, Damian Roland, Akash Deep

**Affiliations:** 1 Evelina London Children’s Hospital, London, UK; 2 Clinical Analytics, Guy's and St Thomas’ NHS Foundation Trust, London, UK; 3 Department of Biostatistics & Health Informatics, King's College London, London, UK; 4 Health Sciences, University of Leicester, Leicester, UK; 5 Paediatric Emergency Medicine Leicester Academic (PEMLA) Group, University Hospitals of Leicester NHS Trust, Leicester, UK; 6 Paediatric Intensive Care, King’s College Hospital, London, UK

**Keywords:** Machine Learning, Child Health

## Abstract

**ABSTRACT:**

**Background:**

Early identification of children at risk of sepsis in emergency departments (EDs) is crucial for timely treatment and improved outcomes. Existing risk scores and criteria for paediatric sepsis are not well-suited for early diagnosis in ED.

**Objective:**

To develop and evaluate machine learning models to predict clinical interventions and patient outcomes in children with suspected sepsis.

**Design:**

Retrospective observational study.

**Setting:**

ED of a tertiary care hospital, UK.

**Patients:**

Electronic health records of children <16 years of age attending between 1 January 2018 and 31 December 2019. Patients presenting with minor injuries were excluded.

**Methods:**

Prediction models were developed and validated, using 15 key predictors from triage and post-blood test data. XGBoost, the best-performing machine learning model, integrated these predictors with triage note information extracted via Natural Language Processing.

**Main outcomes:**

(1) Administration of antibiotics; (2) critical care: antibiotics with fluid resuscitation above 20 mL/kg or non-elective mechanical ventilation; (3) serious infection: hospital admission for antibiotics >48 hours.

Model performance was evaluated using area under the receiver operating characteristic curve (AUC), likelihood ratios and positive and negative predictive values.

**Results:**

Triage model: predicted antibiotics at triage (n=35 795; 3.2% with outcome) with an AUC of 0.80 (95% CI 0.76 to 0.84).

Antibiotic model: predicted antibiotics post-blood tests (n=4700; 24.2%) with an AUC of 0.78 (95% CI 0.73 to 0.81).

Critical care model: predicted critical care (n=4700; 3.3%) with an AUC of 0.78 (95% CI 0.72 to 084).

Serious infection model: predicted serious infection (n=4700; 9.4%) with an AUC of 0.76 (95% CI 0.71 to 0.81).

Key predictors included triage category, temperature, capillary refill time and C reactive protein.

**Conclusion:**

Machine learning models demonstrated good accuracy in predicting antibiotic use following triage and moderate accuracy for critical care and serious infection. Further development and external validation are ongoing.

**WHAT IS ALREADY KNOWN ON THIS TOPIC**

- Current paediatric sepsis criteria are not designed for early identification or risk prediction in emergency departments, which limits timely recognition and intervention.

**WHAT THIS STUDY ADDS**

- Machine learning models using routinely collected electronic health records can accurately predict treatment decisions for children with suspected sepsis, supporting timely and targeted clinical decision-making in the paediatric emergency department.

**HOW THIS STUDY MIGHT AFFECT RESEARCH, PRACTICE OR POLICY**

- The findings provide a foundation for integrating machine learning into real-time clinical decision support for paediatric sepsis in emergency settings.
- Multicentre prospective validation is underway to assess generalisability and pathways for clinical implementation.

## Introduction

 Sepsis is a leading cause of morbidity, mortality and healthcare utilisation for children worldwide.^1^ Early recognition is crucial for initiating timely resuscitation and management for optimising outcomes for children with sepsis.^2^

Sepsis at present, however, still lacks a gold standard diagnostic test.^3 4^ The new Phoenix Sepsis Criteria (PSC)^5^ were derived and validated to predict mortality in children with suspected or confirmed infection. These new data-driven criteria for paediatric sepsis and septic shock were based on measures of organ dysfunction and showed improved performance compared with previous paediatric sepsis criteria.^6^ However, the PSC definitions present new challenges for clinical practice, as the criteria were not intended for early identification of children at risk for sepsis and septic shock.^7^,^9^

Our study aims to address these challenges by designing models that predict clinical interventions and patient outcomes for sepsis in children attending the paediatric emergency department (ED). The models use machine learning (ML) to predict the administration of antibiotics or resuscitative care and aim to prevent physiological decompensation and organ dysfunction. This method is particularly valuable to clinicians, as initial resuscitative care is more common than the development of multiorgan dysfunction due to sepsis in ED.^10^

The application of ML to electronic health records (EHRs) offers the ability to capture complex nonlinear relationships between predictors and outcomes with unprecedented predictive accuracy.^510^,^13^ To our knowledge, no prediction models for sepsis have been successfully implemented into clinical practice in EDs.

We developed prototype ML models to predict pragmatic interventions for sepsis that would be valuable to support real-world decision-making by clinicians in ED. These models were developed as Phase 1 of the Artificial Intelligence Sepsis Tracking Online (AiSEPTRON) programme.^14^ Further development and external validation of these prototype models are underway as a separately funded project.

## Methods

### Study design and setting

A retrospective, observational cohort study was undertaken within the ED of St Thomas’s Hospital (STH), a tertiary care institution in the UK. STH is not a major trauma centre (MTC); paediatric major trauma patients (eg, collisions, haemorrhage, stab and gunshot wounds, etc) are usually transferred directly to designated MTCs for stabilisation and definitive management. Data collection lasted 2 years, from 1 January 2018 to 31 December 2019. The study was funded by the National Institute for Health Research, UK (ref: 203295) and approved by the Health Research Authority (ref: 263832).

### Participants

Eligible participants were children aged under 16 years who visited the ED within the study period. Children presenting with minor injuries or trauma (eg, fractures, lacerations, soft tissue injuries, etc) were excluded.

### Data collection

Consecutive patient records were retrieved from the hospital’s Enterprise Data Warehouse (EDW), a comprehensive repository aggregating clinical data across various platforms, including Symphony, the patient activity database and iClinicalManager (Electronic Patient Record)—key EHR systems used within the ED. Specific data points relevant to the study’s criteria were extracted from the EDW by applying Structured Query Language and were used to create both the development and evaluation datasets.

### Outcome measures

The outcome measures were framed to align with the *Improving Pediatric Sepsis Outcomes* (IPSO) sepsis and IPSO critical sepsis cohort criteria,^15^ which define sepsis based on treatments and interventions. This approach aimed to identify patients who received sepsis treatment without requiring a confirmed diagnosis of infection-related organ dysfunction (see online supplemental eTable 1).

We assessed three outcomes, all incorporating antibiotic administration as part of the paediatric sepsis care bundle delivery, which included blood tests, microbiology and antibiotic delivery via intravenous, intramuscular or intraosseous routes.

We assessed three outcomes, all incorporating antibiotic administration as part of the paediatric sepsis care bundle delivery, which included blood tests, microbiology, and antibiotic delivery via intravenous, intramuscular, or intraosseous routes.

*Outcome 1*: administration of antibiotics.

*Outcome 2 (critical care):* administration of antibiotics and fluid resuscitation above 20 mL/kg (or three or more fluid boluses) or non-elective mechanical ventilation.

*Outcome 3 (serious infection):* hospital admission for antibiotics extending beyond 48 hours.

The inclusion of prolonged antibiotic therapy aimed to capture varying degrees of sepsis severity. Children admitted for more than two calendar days of intravenous antibiotics are typically considered by paediatricians to have a serious or invasive bacterial infection or to have had positive bacteriology.

### Candidate predictors

We considered 15 predictors routinely collected at ED attendance, including demographic information (age, gender), arrival mode (walk-in vs ambulance), vital signs, triage category, hospital admission or discharge status and laboratory tests such as full blood counts and C reactive protein levels (CRP); details of all available predictors are shown in online supplemental eTable 2. Paediatric blood cultures, although often performed, have a notoriously low yield, with aetiological agents frequently not identified, even in cases of sepsis.^16^ Moreover, we excluded blood culture results as they are not immediately available to ED clinicians at the point of decision-making.

### Sample size

We calculated the minimum sample sizes for the models using R (V.1.1.3)^17^ and UK-based sepsis outcome prevalence^18 19^ as detailed in table 1.

**Table 1:** Sample size calculation

Outcome	Model 1	Model 2	Model 3	Model 4
	Systemic antibiotic post-triage	Systemic antibiotic postblood test	Systemic antibiotic + critical care	Systemic antibiotic + admission >2 days
Outcome prevalence (%) *	1 (18)	21.6 (19)	3†	9‡
C-statistic (AUC)	0.8	0.8	0.8	0.8
Predictor parameters	25	15	15	15
Minimum sample size	16 666	630	3386	1248

* UK-specific prevalence estimates were obtained by Gomes S through direct correspondence with Borensztajn D, based on data reported in Borensztajn *et al*^19^

† Prevalence estimated at 3% based on a priori pilot data; reported range in Borensztajn *et al*.^19^: 1.74%–9.9%.

‡ Prevalence estimated at 9% based on a priori pilot data; reported prevalence in Borensztajn *et al*.^19^: 18.6%.

AUC, area under curve.

### Data cleaning

Two distinct datasets were created: the triage dataset (patients assessed at triage) and the blood test dataset (a subset of patients who subsequently underwent blood tests). Five members of the clinical care team reviewed the data. Using individual patient EHRs, they excluded ineligible cases from the datasets. Examples of excluded cases were patients with minor injuries that were inaccurately coded, those who left the department before assessment or patients older than 16 years. The team also examined scanned medical records to collect detailed information for the blood test group. This included specifics such as the duration of intravenous antibiotic administration, receipt and volume of fluid boluses, ventilation details, paediatric intensive care unit (PICU) admissions, in-hospital deaths and any missing vital signs or blood test results.

### Missing data

For both datasets, patients with missing information on >80% of the 15 candidate predictors were excluded, to maintain data integrity. Vital signs presenting out-of-range values, indicative of human error at triage, were removed. However, other relevant predictor variables for these patients were retained in the dataset. Subsequent imputation steps were used to impute missing or deleted variables (see online supplemental eTable 3).

A new category of ‘unknown’ was created for triage category missing values. Missing values for the Glasgow Coma Scale (GCS) or Alert, response to Voice, Pain or Unconscious (AVPU) score were assumed to be alert. Missing triage notes were left empty.

Missing numeric predictors were addressed using a single imputation approach based on Scikit-learn’s IterativeImputer.^20^ This implementation employs the multiple imputation by chained equations (MICE) framework,^21^ which sequentially models each variable with missing values as a function of the other variables in the dataset. Specifically, we used a Bayesian ridge regression model as the predictive estimator within the MICE process, ensuring that the imputations captured complex relationships between variables. The single imputation was chosen to retain the inherent randomness of the process while maintaining computational efficiency and model simplicity.

For the validation and training datasets in both the triage and blood test groups, all numerical variables (vital signs only) were completed using imputation, ensuring that no predictors were missing. However, imputation was not applied to the corresponding test sets. The test sets hence represent patients with original, complete data.

### Feature engineering

Numeric data was used in its raw form without normalisation or standardisation. Triage category codes were encoded using one-hot encoding to represent the different categories. Binary indicators were created for specific categorical features, including ambulance arrivals, capillary refill time (CRT) (>2 s), AVPU scale (dichotomised as alert vs not) and an adapted systemic inflammatory response syndrome (SIRS) alert flag to denote their presence or absence. (online supplemental eTable 4).

In the triage dataset, free-text triage notes were converted into numerical features using natural language processing (NLP). This process leveraged a pretrained transformer-based model developed by Chang *et al*,^22^ which predicted chief complaints from triage notes using a streamlined bidirectional encoder representations from transformers architecture, optimised for efficiency and contextual understanding of clinical text. We refined this approach by training this model on an expanded dataset of two million triage notes, tailoring it to our specific outcome: antibiotic administration. This adaptation enabled the model to extract and encode clinically relevant linguistic patterns into 10 numerical features, capturing key elements of triage notes associated with antibiotic decision-making. Full implementation details are provided in the online supplemental methods.

### Model development

We developed four predictive models targeting the three clinical outcomes mentioned previously:

1. *Triage model (model 1):* predicts the administration of antibiotics using information available at triage.
2. *Antibiotic model (model 2):*predicts the administration of antibiotics following blood testing in the ED.
3. *Critical care treatment model (model 3):* predicts the administration of antibiotics and significant interventions (fluid bolus >20 mL/kg or non-elective ventilation).
4. *Serious infection model (model 4):* predicts the administration of antibiotics and an extended hospital stay exceeding 2 days.

Model 1 was built using the triage dataset, while models 2–4 used the blood test dataset. Four ML algorithms were evaluated for each model: logistic regression,^23^ random forests,^24^ XGBoost^25^ and deep neural networks.^26^

### Feature selection

To optimise the input features, stepwise feature selection was employed using XGBoost models for each dataset. Predictors were sequentially added, and their contributions were assessed based on a minimum increase of 0.05 in the area under the receiver operating characteristic (ROC) curve (AUC). Features that met this criterion were retained for final model training.

### Model training

Training and validation datasets were used to train the models, incorporating early stopping to prevent overfitting where applicable (eg, XGBoost and neural networks). Hyperparameter tuning was conducted through a randomised grid search.

To address class imbalance, hyperparameters such as XGBoost’s scale-positive weight were fine-tuned during the grid search. Although methods like synthetic minority oversampling technique were considered, they were ultimately not employed due to their propensity to increase false positive rates.^27^

Fairness was ensured by using deidentified data and consecutively including all patients in the training, validation and test sets. This minimised the risk of introducing bias from demographic characteristics such as gender, age, ethnicity or socioeconomic status.

### Model evaluation

Model predictions were calculated using the output probabilities generated by the predict_proba function of the Scikit-learn library.^20^ The classification thresholds for the model’s output predictions were set to achieve a sensitivity of 72.7%, selected based on prior evidence indicating a similar sensitivity for physician judgement in identifying severe sepsis and septic shock.^28^ Our goal was to develop models that align with real-world clinical decision-making, with the rationale that integrating algorithm alerts with physician assessment in future practice could significantly improve both sensitivity and specificity.^28^ Model performance was assessed in terms of AUC, sensitivity, specificity, likelihood ratios, positive and negative predictive values (PPV, NPV) and calibration slope. BCa Bootstrap CIs (10 000 iterations) were calculated using R (V.4.3.2) with packages pROC (V.1.18.5)^29^ and boot (V.1.3–28),^30^ with exact CIs on proportions generated through R’s binom.test function.

### Patient and public involvement

A five-member young persons advisory group played a vital role in the study and provided essential oversight throughout the research process. They contributed to the study’s design, refining research questions, providing input into the data collection methodologies, ensuring ethical considerations in data usage, reviewing analyses and participating in dissemination activities (further details online supplemental PPI).

## Results

**Figure 1:**
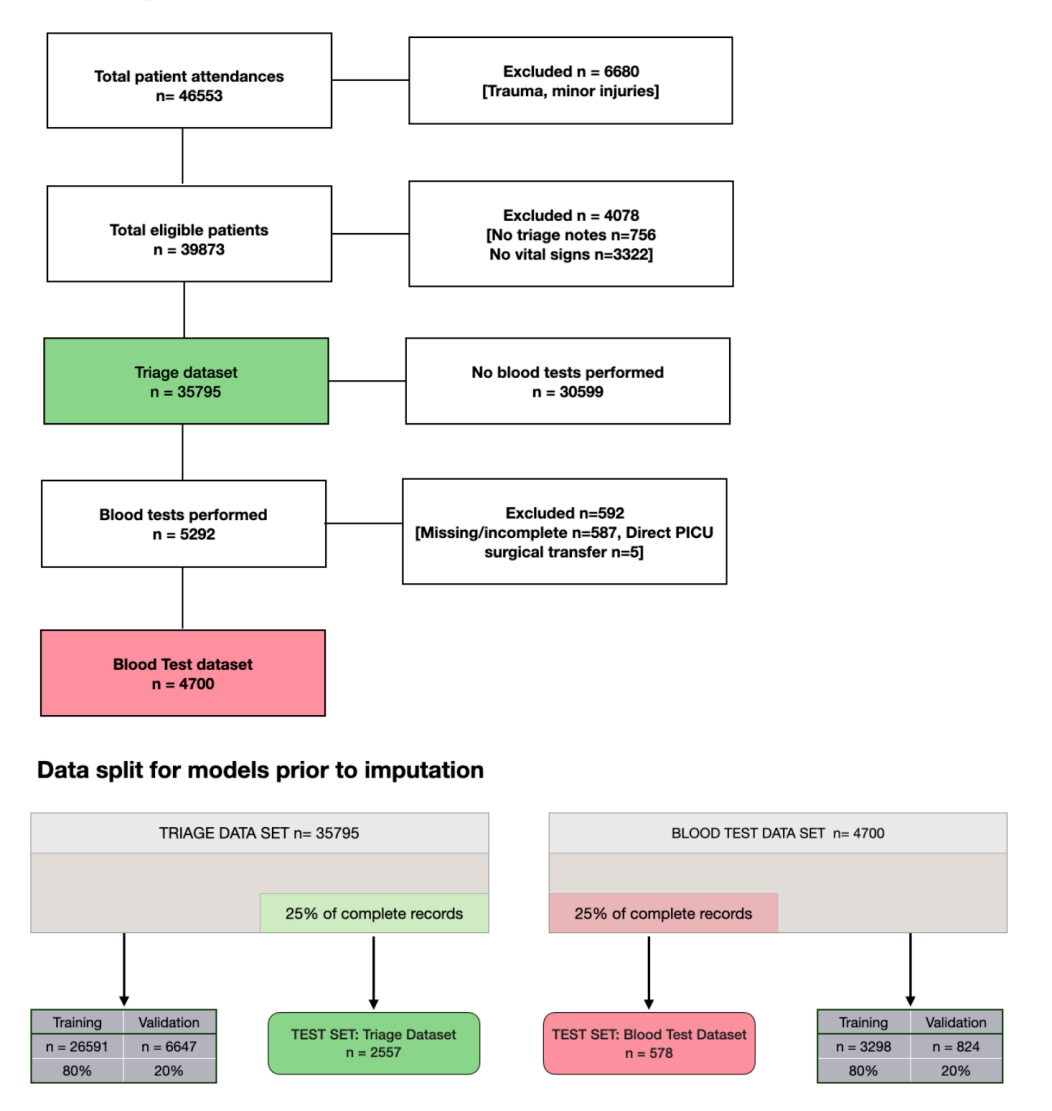
Flow diagram of data processing and splitting before imputation, illustrating the training, validation and test sets for the triage and blood test datasets. PICU, paediatric intensive care unit.

Between 1 January 2018 and 31 December 2019, the ED recorded 46 553 patients, including all cases of trauma and minor injuries (figure 1).

### Data sets

The triage dataset (n=35 795 patients) was divided into three subsets: training (n=26 591, 74%), validation (n=6647, 19%) and test (n=2557, 9%). The blood test dataset (n=4700 patients) was split into: training (n=3298, 70%), validation (n=824, 18%) and test sets (n=578, 12%) as shown in figure 1.

Of the total patients, including injuries, that attended ED (n=46 553), 4700 (10.1%) had blood investigations for suspected infection. 1138 (2.4%) received intravenous antibiotics (Outcome 1), 155 (0.3%) received critical care treatment (Outcome 2), and 443 (0.95%) received intravenous antibiotics and were hospitalised for more than 2 days (Outcome 3) (table 2).

**Table 2:** Age distribution and outcomes

Age group	Patients triaged (n)	Patients that had blood tests (n)	Outcome 1: patients that received intravenous antibiotics (n)	Outcome 2: patients that received intravenous antibiotics and critical care treatment (n)	Outcome 3: patients that received intravenous antibiotics with LOS >2 days (n)
	**Triage dataset**	**Blood test dataset**			
0 days–1 week	405	90	30	4	19
>1 week–1 month	982	228	79	14	54
>1 month–1 year	12 258	1431	432	83	153
≥2–5 years	10 465	1123	287	26	94
≥6–12 years	8795	1247	232	22	91
≥13–16 years	2890	581	78	6	32
Total	35 795	4700	1138	155	443

LOS, length of stay.

The triage dataset (n=35 795), 1138 (3.2%) received intravenous antibiotics (Outcome 1). From the blood test dataset, representing patients investigated for infection (n=4700), 1138 (24.2%) received intravenous antibiotics (Outcome 1), 155 (3.3%) received critical care (Outcome 2) and 443 (9.4%) had over 2 days of admission for intravenous antibiotics (Outcome 3) (table 2). 45 patients were admitted to PICU, and there were three fatalities.

### Model performance

**Figure 2:**
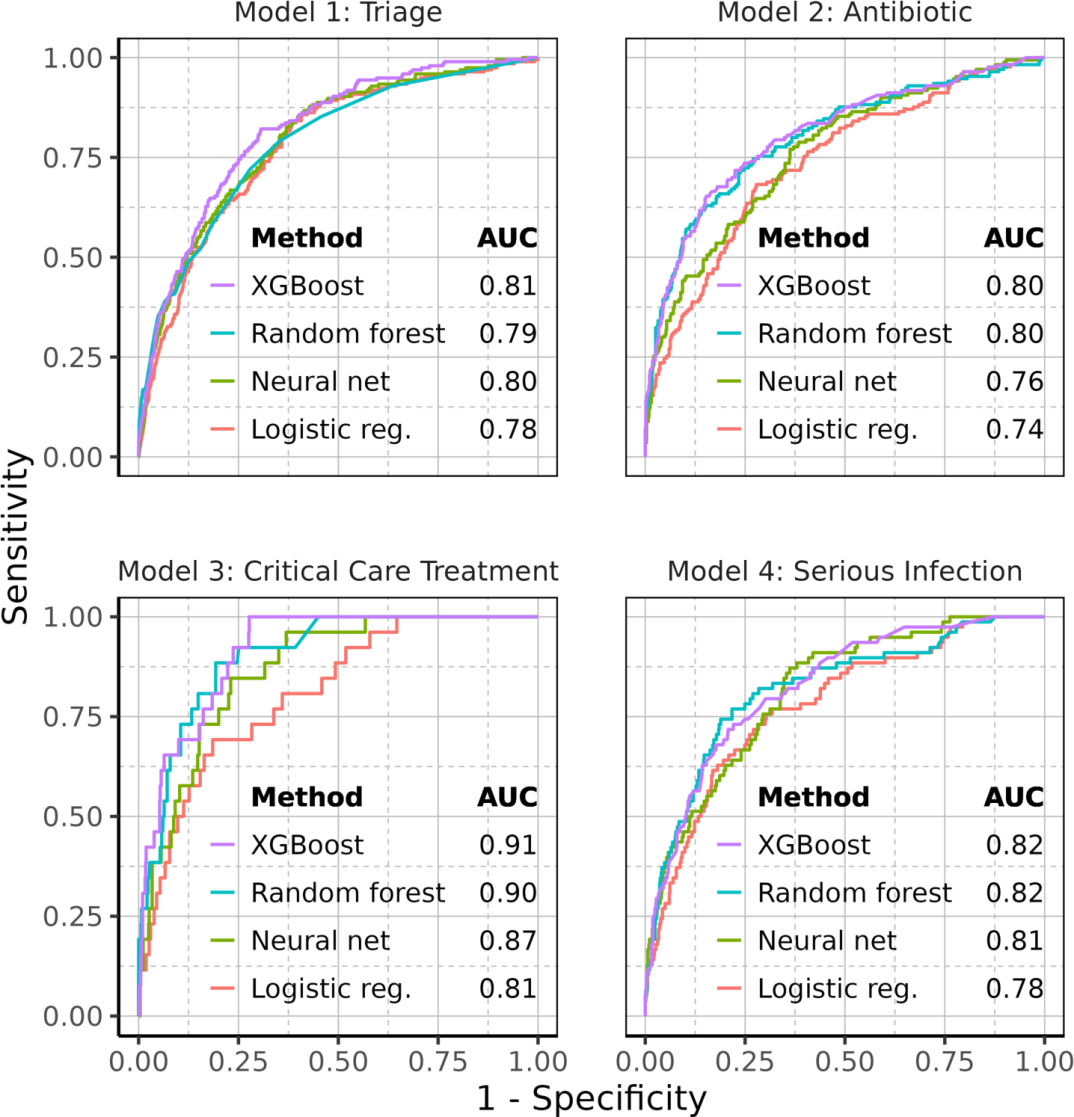
Validation AUCs for machine learning methods. Receiver operating characteristic curves for all four machine learning models, with corresponding area under the curve (AUC) values.

XGBoost demonstrated superior performance compared with alternative methods, leading to its selection as the definitive approach for evaluation on the test sets (figure 2).

The hyperparameters used in the final XGBoost model, optimised through a random grid search process, are summarised in table 3.

**Table 3:** Final XGBoost model hyperparameters derived from the random grid search optimisation

Hyperparameter	Model 1	Model 2	Model 3	Model 4
max_depth	4	3	5	2
n_estimators	20	15	79	68
learning_rate	0.469	0.4485	0.7417	0.5321
reg_alpha	0.2426	0.6708	2.0537	1.4188
reg_lambda	1.6287	3.4194	1.716	2.5734
gamma	0.05	8.523	4.916	4.561
scale_pos_weight	3.679	2.259	4.495	2.181
callbacks	(early_stop)	(early_stop)	(early_stop)	(early_stop)

**Figure 3:**
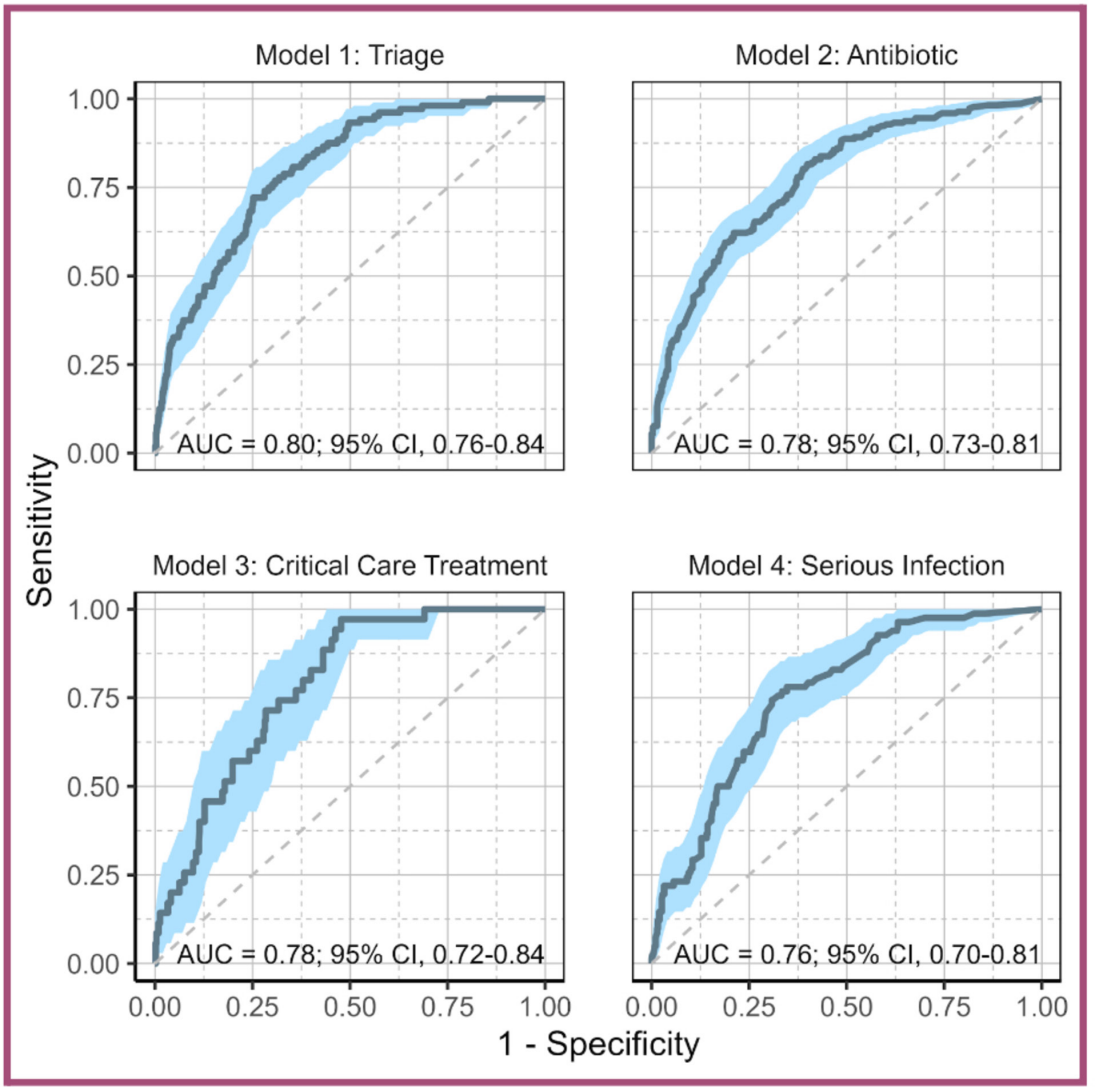
Test set AUCs. Receiver operating characteristic curves for all four models, displaying area under the curve (AUC) values with 95% CIs.

Figure 3 presents the test set ROC curves and their corresponding AUCs and 95% CIs for each model. The triage model (which used the largest training set (n=26 591) performed well and achieved an AUC of 0.80. The remaining three models achieved AUCs between 0.76 and 0.78.

**Figure 4:**
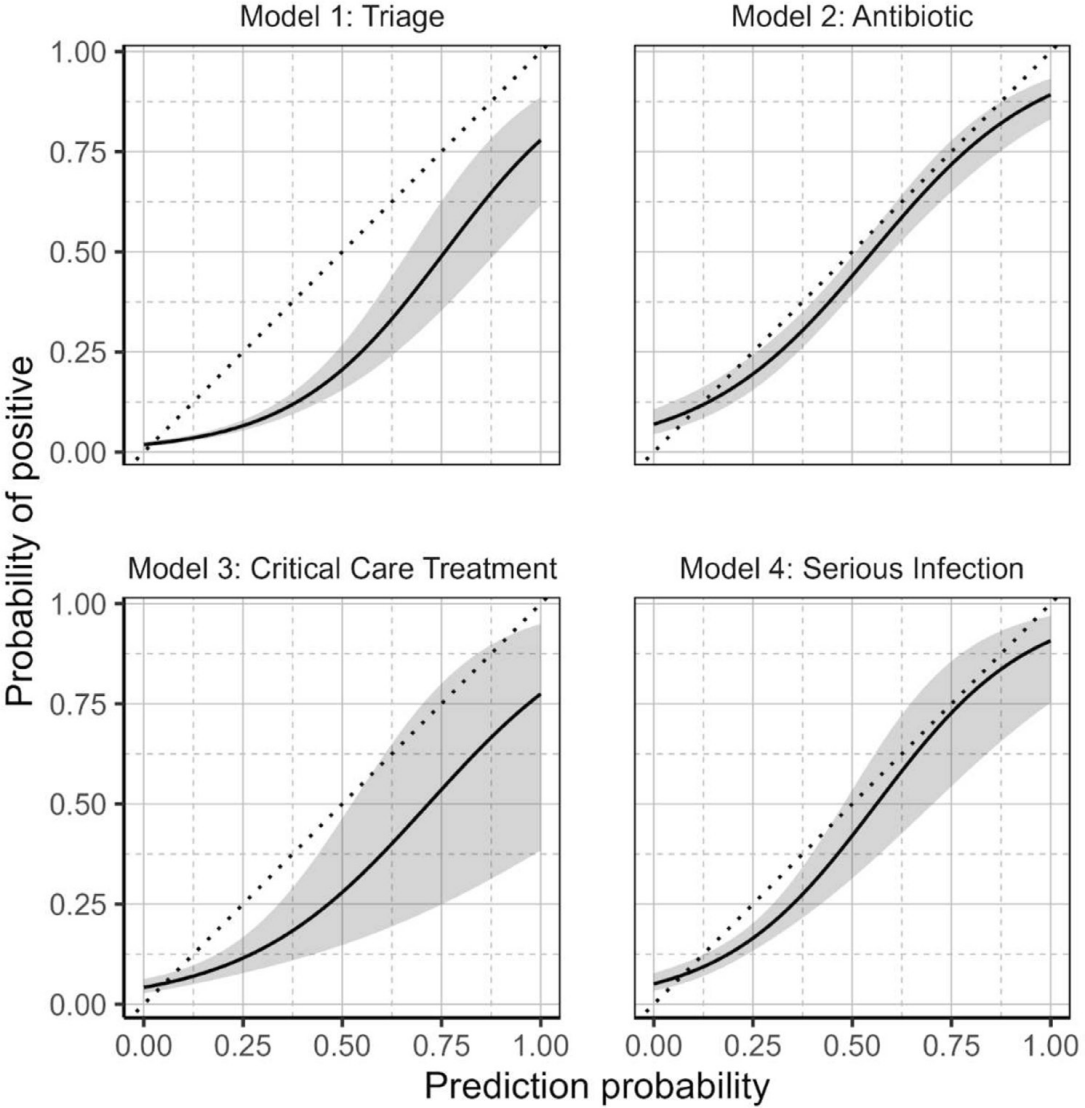
Calibration curves for the four XGBoost models. Calibration plots using binomial generalised additive models (GAMs) to map predicted probabilities to actual outcomes for the four machine learning models. The shaded area represents the 95% CI. GAMs were fitted using the ‘gam’ package in R.

Figure 4 presents calibration slopes for the four models; these show a tendency to overestimate probabilities in the triage and critical care treatment models.

The specificities of the four models ranged between 65% and 72% (table 4).

**Table 4:** Model performance

Model	Description	Prevalence in test setpositives/total = %	AUC	Specificity	PPV	NPV	LR+	LR-
Model 1	Triage model	104/2557=4%(3% to 5%)	80 %(76 to 84)	72 %(62 to 77)	10 %(7 to 13)	98 %(98 to 99)	2.59(1.90 to 3.13)	0.38(0.36 to 0.44)
Model 2	Antibiotic model	222/578=38%(34% to 42%)	78 %(73 to 81)	65 %(59 to 73)	57 %(51 to 64)	79%(76 to 82)	2.10(1.77 to 2.67)	0.42(0.38 to 0.46)
Model 3	Critical care treatment model	33/578=6%(4% to 8%)	78 %(72 to 84)	68 %(56 to 78)	13 %(8 to 20)	97%(96 to 98)	2.30(1.63 to 3.34)	0.40(0.35 to 0.49)
Model 4	Serious infection model	82/578=15%(11% to 17%)	76 %(71 to 81)	69 %(55 to 75)	28 %(21 to 36)	94%(92 to 95)	2.38(1.63 to 2.96)	0.39(0.36 to 0.49)

Performance of each of our models, if a sensitivity of 72.7% is targeted (to match that of a clinician).

In brackets, 95% BCa bootstrap CIs (for prevalence, Clopper-Pearson exact proportion CIs).

AUC, area under curve; LR, likelihood ratios; NPV, negative predictive value; PPV, positive predictive value.

The performance of the triage and critical care treatment models was diminished due to their low prevalence rates of 4% and 6%, respectively, alongside moderate rates of false positives, at 28% and 32%, respectively. Consequently, the PPV was low, at 10% for the triage model and 13% for the critical care treatment model.

### Features of importance

From the final XGBoost models, importance scores for each feature were extracted. From over 30 available predictors extracted from the EHRs (online supplemental eTable 2), 15 features were chosen. This was calculated by using the gain from every feature created in each training set, measuring the average improvement in loss made by each feature (table 5).

**Table 5:** Features of importance

Feature	Datatype	Ranking (importance)			
		Model 1: triage	Model 2: antibiotic	Model 3: CCT	Model 4: SI
Age (days)	Continuous	3 (0.065)	7 (0.064)	10 (0.044)	3 (0.125)
Triage category	Categorical	1 (0.270)	5 (0.109)	3 (0.129)	5 (0.115)
Oxygen saturation	Continuous	11 (0.038)	13 (0.000)	4 (0.083)	12 (0.000)
Respiratory rate	Continuous	16 (0.027)	8 (0.057)	7 (0.062)	9 (0.046)
Systolic blood pressure	Continuous	17 (0.026)	3 (0.119)	9 (0.050)	7 (0.073)
Heart rate	Continuous	9 (0.043)	2 (0.153)	2 (0.163)	4 (0.119)
Temperature	Continuous	2 (0.072)	1 (0.203)	6 (0.063)	2 (0.178)
Capillary refill time	Binary	6 (0.046)	14 (0.000)	1 (0.179)	13 (0.000)
Alertness flag	Binary	21 (0.000)	15 (0.000)	15 (0.000)	15 (0.000)
SIRS flag	Binary	19 (0.000)	12 (0.000)	14 (0.000)	10 (0.000)
Ambulance flag	Binary	20 (0.000)	10 (0.027)	13 (0.029)	14 (0.000)
Platelet count	Continuous	–	11 (0.023)	12 (0.034)	8 (0.056)
White blood cell count	Continuous	–	6 (0.097)	11 (0.042)	6 (0.100)
Neutrophil count	Continuous	–	9 (0.034)	8 (0.055)	11 (0.000)
C reactive protein level	Continuous	–	4 (0.114)	5 (0.068)	1 (0.187)
NLP output 1	Continuous	12 (0.038)	–	–	–
NLP output 2	Continuous	8 (0.045)	–	–	–
NLP output 3	Continuous	13 (0.037)	–	–	–
NLP output 4	Continuous	5 (0.056)	–	–	–
NLP output 5	Continuous	4 (0.061)	–	–	–
NLP output 6	Continuous	7 (0.045)	–	–	–
NLP output 7	Continuous	15 (0.033)	–	–	–
NLP output 8	Continuous	10 (0.039)	–	–	–
NLP output 9	Continuous	18 (0.025)	–	–	–
NLP output 10	Continuous	14 (0.034)	–	–	–

Feature Ranking (importance) (gain) for models 1–4. Gain reflects each feature’s contribution to model performance.

CCT, critical care treatment; NLP, Natural Language Processing; SI, serious infection; SIRS, systemic inflammatory response syndrome.

1. A consistent finding across all models was the inclusion of the triage category as a key feature.
2. The foremost predictor varied among the models: triage category for model 1, temperature for model 2, CRT for model 3 and CRP for model 4.
3. The three features consistently important to all four models were temperature, triage category and HR. In addition, CRP emerged as a common predictor in the top three features across the blood test models (2–4), as this was the group of patients who underwent blood tests.
4. The three lowest ranking features common to models 1–4 were the alertness (GCS/AVPU), SIRS and ambulance flag.

## Discussion

We developed and evaluated ML models for predicting clinical interventions and patient outcomes in children with suspected sepsis. From 15 candidate predictors within the EHRs, we identified the most important features contributing to these predictions.

The introduction of the PSC marked a pivotal step in defining paediatric sepsis by focusing on organ dysfunction.^5^ However, these criteria pose several challenges to widespread implementation. First, the criteria had a distinct bias, favouring a PICU-based consensus as only 15% of respondents in the global survey were based in ED. 80% of paediatric patients with sepsis are known to present initially to ED or standard inpatient care settings.^31^ Second, the PSC was not designed for early diagnosis or sepsis risk prediction. The criteria are unsuitable as screening or early warning tools for paediatric sepsis.^7^ Third, the criteria were developed without data from the UK or Europe; of note, many of the included criteria (eg, lactate, coagulation) are not routinely collected from patients with infection in the UK EDs. Combined, these factors limit the generalisability and uptake of the new criteria for many EDs.

This study is unique in concentrating on the most subjective part of the sepsis diagnostic pathway: the cohort of children categorised as ‘suspected infection’ (ie, patients who undergo microbiological testing and receive systemic antibiotics). This group constitutes the biggest challenge for diagnosis and for planning interventions, prior to the onset of organ dysfunction. Our prediction models aim to address this gap targeting this vulnerable group, using outcome measures that are pragmatic and would be useful to clinicians to guide timely interventions. This approach of assigning practical, actionable outcome labels as ground truths for training ML models was acknowledged to be an efficient method for providing actionable predictions.^32 33^

Considering the total ED population (n=46 553), the prevalence of patients that received intravenous antibiotics (Outcome 1) was 2.4% (1138), 0.3% (155) for critical care (Outcome 2) and 0.95% (443) for serious infection (Outcome 3). The critical care and serious infection groups are patients important to clinicians, representing children with the highest risk of disease progression and organ dysfunction.

3.3% (155 of 4700) of those who had blood tests and 13.6% (155 of 1138) of those who were administered intravenous antibiotics received critical care treatment (Outcome 2).

9.4% (443 of 4700) of all patients who had blood tests and 38.9% (443 of 1138) of those who received intravenous antibiotics had hospital stays over 2 days (serious infection, Outcome 3).

Our results emphasise the complexities involved in predicting sepsis in real-world clinical environments. The low prevalence of sepsis outcomes highlights the significant challenge in achieving an optimal balance between sensitivity and specificity across varied patient presentations. This was demonstrated by moderate false positive rates, leading to low positive PPV for the triage model (28% false positives, 10% PPV) and critical care treatment model (32% false positives, 13% PPV).

The triage model, with an AUC of 0.8, has the potential for substantial impact on clinical practice as a screening tool for early risk identification for sepsis. Subsequent to blood test investigations for suspected sepsis, the results integrate into the antibiotic, critical care and serious infection models. Both the serious infection model (AUC: 0.76) and critical care model (AUC: 0.78) achieved moderate accuracy. The balance between sensitivity and specificity, alongside the high NPV, positions the models as useful adjuncts, offering a range of tools that could enhance clinical decision-making, facilitating the determination of appropriate interventions. There was, however, some evidence of miscalibration, with a tendency to overestimate risks in some models (figure 4). The model outputs were designed to represent the probability of predicting the respective outcomes. However, in order for these outputs to be interpreted as true probabilities, additional recalibration of the model would be required.

The models developed spanning triage predictions to critical care needs underscore the value of temperature, triage category, heart rate and CRP as important predictors of sepsis outcomes. Notably, the consistency across all models in identifying these variables of significance reinforces their clinical relevance in sepsis prediction. Furthermore, the commonality of heart rate, temperature, CRP, triage category, blood pressure and age across three of the models (models 2–4) highlights the relevance of these indicators in assessing sepsis risk using available blood test results. The alertness flag (GCS/AVPU) and platelet count performed poorly, suggesting that the majority of patients at risk of serious infection in the ED had not progressed to neurological compromise or organ dysfunction.

Past guidelines and screening pathways using clinical variables and their threshold values^34^ have lacked accuracy.^35^ However, integrating composite analyses that consider a diverse range of parameters has been validated as successful.^36 37^ ML models have the ability to leverage implicit—but yet unrecognised—relationships in clinical data,^32 33^ forming non-linear relationships between multiple clinical variables and outcomes. Studies have shown the importance of combining ML tools with physician judgement to maximise predictive accuracy.^38^ Human-artificial intelligence collaboration will be vital for successful clinical implementation and building trust among patients and clinicians.^39^

The future of paediatric sepsis care and research is inevitably digital.^40^ The authors believe that advanced computational methods, particularly ML, incorporating a broad range of clinical variables, will facilitate the early recognition of sepsis risk more efficiently than traditional risk criteria scoring systems.

### Strengths and limitations

The main strength of the study is the large cohort of paediatric ED attendances and the range of data used to build the models. We acknowledge several limitations: first, the study was conducted at a single centre. Hence, the results might not be universally applicable to other EDs that serve different patient populations.

Second, the models were developed to identify infection-driven deterioration rather than physiological changes associated with major trauma. Including major trauma cases could have introduced confounding variables, given the distinct pathophysiology and clinical management of trauma versus sepsis. Therefore, the prediction models were not designed for use in major trauma patients.

Third, the retrospective data set had an acceptable number of missing clinical data. We addressed this challenge by manual data entry and imputation. However, it is important to note that imputed datasets were solely used for the training and validation sets. Missing blood test results were not imputed.

Fourth, the retrospective dataset did not have adequate numbers for patients who received vasoactive agents or had PICU admissions or in-hospital deaths. Known predictors like lactate and coagulation were not routinely performed. Hence, it was not possible to incorporate these variables into the models or use the Phoenix criteria.

Fifth, the models were developed using vital signs available at triage, the only observations recorded electronically. Manual collection of all serial observations from scanned medical notes was logistically impractical. The inclusion of serial observations leading up to interventions could have enhanced precision.

Finally, the models showed some evidence of miscalibration in test data, with a tendency to overestimate outcome risk. At this stage, recalibration was deemed unnecessary as it would not influence the reported performance metrics. Further, the prototype models were not yet considered ready for clinical implementation. Causes of miscalibration will be investigated and addressed in future phases of the AiSEPTRON programme.^11^

In conclusion, this study developed and evaluated ML models, marking a significant milestone as the first to implement pragmatic outcomes for predicting clinical interventions and patient outcomes in children with suspected sepsis in ED. Further multicentre prospective evaluation of these models is underway, with a planned pathway for integration into clinical practice, to ultimately improve outcomes for children with sepsis.

## Supplementary material

10.1136/bmjpo-2024-003273 online supplemental file 1

## Data Availability

Data are available upon reasonable request.
