# Supplementary material for: Predicting sepsis treatment decisions in the paediatric emergency department using machine learning: the AiSEPTRON study
Source: BMJ Paediatr Open. 2025 May 14;9(1):e003273. doi: 10.1136/bmjpo-2024-003273 (PMC12083314; doi:10.1136/bmjpo-2024-003273)
Supplement: online supplemental file 1 [file bmjpo-9-1-s001.docx]

**ONLINE SUPPLEMENT**

**CONTENTS**

**eTable 1:** Improving Pediatric Sepsis Outcomes (IPSO) sepsis and IPSO critical sepsis cohort criteria

**eTable 2:** Example extract of raw dataset

**eTable 3:** Missing Data: data types and proportions missing or out of range of each feature in the two cleaned datasets.

**eTable 4:** SIRS Alert Flag

**Methods:** Natural Language Processing Methods

**Methods:** Patient and Public Involvement (PPI)

**eTable 1:** Improving Pediatric Sepsis Outcomes (IPSO) sepsis and IPSO critical sepsis cohort criteria

| **Improving Pediatric Sepsis Outcomes (IPSO) sepsis and IPSO critical sepsis cohort case criteria** | |
| --- | --- |
| **Term** | **Definition** |
| IPSO suspected infection | Hospitalized patients or ED patients designated for admission who had a blood culture collected and an antibiotic administered within 24 hours of each other |
|  |  |
| IPSO sepsis | Hospitalized patients or ED patients who met ≥1 of the following 8 criteria |
|  | A bedside assessment positive for sepsis and treatment of sepsis * |
|  | A positive huddle for sepsis |
|  | Use of an electronic medical record order set for severe sepsis or use of an electronic medical record order set for sepsis that included treatment of sepsis * |
|  | ICU admission and treatment of sepsis * |
|  | Collection of a venous or arterial lactate level and treatment of sepsis* |
|  | Administration of a vasopressor and treatment of sepsis * |
|  | Use of ICD-10 billing codes R65.20 or R65.21, or use of other specified sepsis ICD-10 billing codes and treatment of sepsis * |
|  |  |
| IPSO critical sepsis | Hospitalized patients or ED patients who met the IPSO sepsis definition and met ≥1 of the following criteria |
|  | Administration of a first antibiotic plus 2 IV fluid boluses (all within 6 h of each other) plus administration of a third IV fluid bolus, administration of a first antibiotic plus 2 IV fluid boluses (all within 6 h of each other) plus administration of a vasopressor, |
|  | Or administration of a first antibiotic plus an IV fluid bolus plus administration of a vasopressor (all within 6 h of each other) |
|  |  |
| * Administration of an antibiotic and an IV fluid bolus plus either a second IV fluid bolus or administration of a vasopressor (all within 6 h of each other) plus a blood culture collected within 72 h of the episode. | |
| ICD-10, International Classification of Diseases, 10th Revision; IV, intravenous. | |

**eTable 2: Example extract of raw dataset**

| **Study ID** | **HospID** | | **AttendanceID** | | **AgeDays** | | **PatientAgeGroup** | | **GenderFLG** | | **ArrivalDT** | | **ArrivalDTS** | | | **TriageDTS** | | **TriageCategoryDSC** | | **TriageNotes** | | | | | **AmbulanceArrivalFLG** |
| --- | --- | --- | --- | --- | --- | --- | --- | --- | --- | --- | --- | --- | --- | --- | --- | --- | --- | --- | --- | --- | --- | --- | --- | --- | --- |
| X1234 | Y1234 | | Z12345 | | 184 | | 3-6 months | | F | | 2019-01-28 00:00:00 | | 2019-01-28 18:04:00 | | | 2019-01-28 18:12:00 | | Other | | gp referral for fever ? cause temp was 38.5 with gp | | | | | 1 |
| X12345 | Y12345 | | Z12346 | | 1242 | | 3-4 years | | M | | 2019-11-25 00:00:00 | | 2019-11-25 16:25:00 | | | 2019-11-25 16:38:00 | | 2 (Orange) | | 1/7 fever up to 39.9. runny nose vomiting x3. mum noticed blood in vomit but also it came from the nose to. drinking ok | | | | | 1 |
| X23456 | Y23456 | | Z12347 | | 475 | | 1-2 years | | M | | 2018-04-01 00:00:00 | | 2018-04-01 11:15:00 | | | 2018-04-01 11:43:00 | | 3 (Yellow) | | In church today had seizure, full body shaking, eye rolling, went purple. Lasted less than 2 min. Fever on LAS arrival to 39.4, had been well ebfore this. Had revious febrile convulsion last year,. | | | | | 1 |
| X34567 | Y34567 | | Z12348 | | 361 | | 1-2 years | | M | | 2018-08-14 00:00:00 | | 2018-08-14 11:50:00 | | | 2018-08-14 11:55:00 | | 2 (Orange) | | Sent by GP for fever of unknown origin - See attached letter, in triage, baby upset crying ++ ,, no WOB. | | | | | 0 |
| X45678 | Y45678 | | Z12349 | | 4510 | | 12-16 years | | M | | 2019-07-19 00:00:00 | | 2019-07-19 20:05:00 | | | 2019-07-19 20:30:00 | | 2 (Orange) | | called 999 as copuldnt walk with pain in legs, 3 days unwell 3 days lump on neck with leg pain getting worse. temperature today | | | | | 1 |
| X56789 | Y56789 | | Z12350 | | 2170 | | 6-7 years | | M | | 2019-07-06 00:00:00 | | 2019-07-06 18:36:00 | | | 2019-07-06 18:50:00 | | Other | | 4/7 Hx fever up to 39.6 at home, cough & coryzal, with increased work of breathing at home. 1 cough induced vomit and abdo pain. Eating and drinking well. | | | | | 1 |
| X67900 | Y67900 | | Z12351 | | 1999 | | 5 years | | F | | 2019-10-30 00:00:00 | | 2019-10-30 12:40:00 | | | 2019-10-30 12:43:00 | | 2 (Orange) | | Fever 40 degrees last night. Called 111 then visited GP today. Fever this morning, pain in stomach, head and throat. 1x vomit, Reduced eating and drinking. BNO 2/7. Dad unsure whether she has passed urine today. | | | | | 0 |
| X79011 | Y79011 | | Z12352 | | 1056 | | 1-2 years | | F | | 2019-06-03 00:00:00 | | 2019-06-03 12:52:00 | | | 2019-06-03 13:21:00 | | 2 (Orange) | | High fever for 2 days. Febrile convulsion in GP. Dad cant think of obvious symptoms. No cough, cold, pain or obvious source. | | | | | 1 |
| X90122 | Y90122 | | Z12353 | | 3656 | | 8-12 years | | M | | 2019-01-10 00:00:00 | | 2019-01-10 11:11:00 | | | 2019-01-10 11:27:00 | | 3 (Yellow) | | 3 day hx of Rt arm pain worse today, had to come home from school, went to GP who adviced to come to dept, has an appt with Dr Inusa tomorrow mum kindly asking if he can see Diamond today, whole arm painfull, no swelling or redness. | | | | | 0 |
| **PrimaryDiagnosis** | | **PrimaryDiagnosisTYPE** | | **NEWSSPO2VAL** | | **NEWSRespVAL** | | **NEWSBPSystolicVAL** | | **NEWSBPDiastolicVAL** | | **NEWSPulseVAL** | | **NEWSTempVAL** | **GlasgowComaScore** | | **Capillary_Refill_Time** | | **ACVPU** | | **Platelets_Results** | **WhiteBlood_Results** | **Neutrophils_Result** | **C_Reactive_Protein_Level_Result** | |
| Urinary tract infection | | Suspected diagnosis | | 100 | | 22 | | 122 | | 81 | | 98 | | 38.1 |  | |  | | alert | | 279 | 19.6 | 12 | 116 | |
| Upper respiratory tract infection | | Suspected diagnosis | | 100 | | 36 | | 128 | | 72 | | 155 | | 39.3 |  | | <2 | | Alert | | 443 | 24.9 | 13.9 | 29 | |
| Seizure : febrile | | Suspected diagnosis | | 99 | | 28 | | 91 | | 64 | | 205 | | 38.2 |  | | <2 | | Alert | | 312 | 20.4 | 16.7 | <1 | |
| Sepsis | | Suspected diagnosis | | 100 | | 46 | | 128 | | 80 | | 158 | | 39 |  | | <2 | | Alert | | 276 | 10.7 | 5.4 | 73 | |
| Sickle cell crisis | | Confirmed diagnosis | | 94 | | 24 | | 119 | | 63 | | 107 | | 38.8 |  | | <2 | | Alert | | 389 | 8.3 | 3.1 | 8 | |
| Measles [ND] | | Suspected diagnosis | | 98 | | 35 | | 98 | | 72 | | 112 | | 37.5 |  | |  | | alert | | 218 | 9.6 | 4.4 | 6 | |
| Upper respiratory tract infection | | Suspected diagnosis | | 98 | | 28 | | 110 | | 60 | | 152 | | 39.4 |  | | <2 | | Alert | | 121 | 10.3 | 8.3 | 45 | |
| Tonsillitis | | Suspected diagnosis | | 98 | | 52 | | 103 | | 75 | | 180 | | 39.7 |  | | 3 | | Alert | | 291 | 23.7 | 19 | 37 | |
| Sickle cell crisis | | Confirmed diagnosis | | 100 | | 26 | | 112 | | 70 | | 81 | | 37.3 |  | | <2 | | Alert | | 348 | 10.9 | 8.3 | 1 | |

**eTable 3: Data types and proportions missing or out of range of each feature in the two cleaned datasets.**

| **Dataset** | **Feature** | **Data type** | **Missing or out of range (%)** | |
| --- | --- | --- | --- | --- |
| **Triage** |  |  |  |  |
|  | Age | Continuous | 0 | 0.00% |
|  | Triage category | Text/discrete categories | 11 | (0.03%) |
|  | Triage notes | Text | 0 | 0.00% |
|  | Systolic blood pressure | Continuous | 24229 | (67.68%) |
|  | Heart rate | Continuous | 825 | (2.30%) |
|  | Respiratory rate | Continuous | 1274 | (3.55%) |
|  | Oxygen saturation | Continuous | 1139 | (3.18%) |
|  | Temperature | Continuous | 2598 | (7.26%) |
|  | Capillary refill time | Discrete | 4208 | (10.77%) |
|  | AVPU score | Discrete | 3057 | (7.83%) |
| **Blood test** | |  |  |  |
|  | Systolic blood pressure | Continuous | 1915 | (40.7%) |
|  | Heart rate | Continuous | 95 | (2.0%) |
|  | Respiratory rate | Continuous | 128 | (2.7%) |
|  | Oxygen saturation | Continuous | 103 | (2.2%) |
|  | Temperature | Continuous | 223 | (4.7%) |
|  | Ambulance flag | Discrete | 0 | 0.0% |
|  | Capillary refill time | Discrete | 140 | (3.0%) |
|  | AVPU score | Discrete | 8 | (0.2%) |
|  | Platelet count | Continuous | 0 | 0.0% |
|  | White blood cell count | Continuous | 0 | 0.0% |
|  | Neutrophil count | Continuous | 0 | 0.0% |
|  | C Reactive Protein Level | Continuous | 548 | (11.7%) |

**eTable 4: SIRS alert flag**

| **Age group** | **Heart rate, Beats/ min** | | **Breaths/ min** | **mm Hg** | **Leucocyte X 10^3/mm** |
| --- | --- | --- | --- | --- | --- |
|  | Tachycardia | Bradycardia | Respiratory rate | Systolic BP | Leucocyte |
| 0 days to 1 wk | > 180 | <100 | >50 | <65 | >34 |
| 1 wk to 1 mo | > 180 | <100 | >40 | <75 | >19.5 or <5 |
| 1 mo to 1 yr | > 180 | <90 | >34 | <100 | >17.5 or <5 |
| 2–5 yrs | > 140 | NA | >22 | <94 | >15.5 or <6 |
| 6–12 yrs | > 130 | NA | >18 | <105 | >13.5 or <4.5 |
| 13 to < 18 yrs | > 110 | NA | >14 | <117 | >11 or <4.5 |
|  | | | | | |
|  |  |  |  |  |  |
| **SIRS Flag** = Either Temperature > 38C or < 36C or abnormal Leucocyte count, plus one trigger from of either of heart rate or respiratory rate or blood pressure, with suspected infection | | | | | |

**Methods**

**Natural language Processing methods**

We aimed to incorporate free-text triage notes into our prediction models using a technique called **transfer learning** [1]. Transfer learning allows a model that has already learned from a large dataset to be adapted for a new task with a smaller dataset. In our study, we used a model developed by Chang et al. [2], trained on over 2 million triage notes.

Chang et al.’s model builds on **BERT** (Bidirectional Encoder Representations from Transformers), a language model developed by Google that processes text by understanding words in context. The version used by Chang et al. was further refined using a large dataset of medical records to predict common diagnoses from triage notes.

In practice, this model converts each triage note into 768 numerical features — a compact representation of the note’s content. However, using all 768 features would add unnecessary complexity and noise, so we applied additional steps to extract only the most relevant information.

To do this, we added two layers to the model:

1. **Feature Reduction Layer:** This layer compressed the original 768 features into just **10 key features** that were most relevant to predicting sepsis-related outcomes.
2. **Prediction Layer:** These 10 features were then passed into a small neural network to help predict sepsis-related outcomes.

The model was trained lightly — just enough to map the NLP features to sepsis outcomes while avoiding overfitting due to our smaller dataset. We then added these 10 features to the XGBoost models used in the study.

However, early experiments showed that including these NLP features in some models (Models 2–4) introduced noise, likely due to the limited dataset size and low number of positive cases. As a result, we decided not to include the NLP features in these models for now, though we plan to revisit this in future work with a larger dataset.

Further refinement of the original BERT model could improve performance, but this was beyond the scope of our current study.

**REFERENCES**

**1)** Azunre, P. Transfer learning for natural language processing. Simon and Schuster. 2021.

2) Chang D, Hong WS, Taylor RA. Generating contextual embeddings for emergency department chief complaints. [published online July 15, 2020]. JAMIA Open. doi: 10.1093/jamiaopen/ooaa022.

**Patient and Public Involvement (PPI)**

A Patient and Public Engagement Involvement Grant [NIHR, EIF:351] funded the creation of a dedicated Young Person’s Advisory Group (YPAG) composed of five research members.

The YPAG contributed to the study's design, refining research questions, providing input into the data collection methodologies, ensuring ethical considerations in data usage, reviewing analyses, and participating in dissemination activities. They provided essential oversight throughout the research process.

Workshops were convened where YPAG members were introduced to concepts of sepsis, artificial intelligence, research methodology and ethics around patient identifiable data.This workshop served as a foundation for their ongoing involvement and allowed them to offer practical input into several key areas:

**Refining Research Questions:** The YPAG reviewed the study's initial research questions, ensuring they were relevant and understandable from a public perspective. They emphasized the importance of identifying sepsis early and highlighted the need for clear communication in any tools developed to support clinical decision-making.

**Input into Data Collection Methodologies:** The YPAG provided practical suggestions regarding the collection and handling of retrospective patient data, particularly regarding consent and transparency. Given the scale of data collection and the use of de-identified records, they proposed strategies for parental awareness, through improved communication, such as displaying posters in public areas of the emergency department and utilizing social media and Trust websites to inform parents about the study.

**Ethical Considerations in Data Usage:** One of the primary ethical concerns raised by the YPAG was the access to identifiable patient data for completing missing records. The group felt that access should be restricted to the key researchers and research nurses only, beyond the direct clinical care team.

**Dissemination Activities:** The YPAG contributed to the development of materials aimed at engaging families, such as study posters and social media campaigns, and advised on creating a study website to improve accessibility and transparency. activities.
