# Supplementary material for: Predicting sepsis treatment decisions in the paediatric emergency department using machine learning: the AiSEPTRON study
Source: BMJ Paediatr Open. 2025 May 14;9(1):e003273. doi: 10.1136/bmjpo-2024-003273 (PMC12083314; doi:10.1136/bmjpo-2024-003273)
Supplement: Supplementary file 2 [file bmjpo-2024-003273.reviewer_comments.pdf]

PEER REVIEW HISTORY

BMJ Paediatrics Open publishes all reviews undertaken for accepted manuscripts. Reviewers are asked to complete a checklist review form and are provided with free text boxes to elaborate on their assessment. These free text comments are reproduced below.

ARTICLE DETAILS

**TITLE (PROVISIONAL)**	Predicting Sepsis Treatment Decisions in the Paediatric Emergency Department Using Machine Learning: The AiSEPTRON Study
**AUTHORS**	Gomes, Sylvester; Dhanoa, Harpreet; Assheton, Phil; Carr, Ewan; Roland, Damian; Deep, Akash

VERSION 1 - REVIEW

**REVIEWER NAME**	***Colin Powell***
**REVIEWER AFFILIATION**	None disclosed
**REVIEWER CONFLICT OF INTEREST**	N/A
**AI DISCLOSURE**	No
**DATE REVIEW RETURNED**	10-Mar-2025

**GENERAL COMMENTS**	This is a fascinating study using AI and machine learning in clinical modelling in predicting various aspects of paediatric sepsis in the ED. The new Pheonix sepsis criteria are not designed for use in the ED and so this is a step towards validating models for the ED population. Congratulations on this work. To the reader who is not au fait with machine learning and AI , it takes some time to understand some sections and potentially could be improved if it was simplified further.<br>This is a single (tertiary) centre retrospective observational study. Two years electronic medical notes analysis from a ED (minor injuries excluded) are used to derive four models for prediction<br><br>1) The abstract should be more explicit about 15 predictive parameters were used for the modelling and these were based on information at triage and information post blood tests.<br>2) Some of the information in your intervention section in your abstract (eg XGBoost, neural networks, and random forests, Natural Language Processing outputs) was unclear to a reader who does not know this territory - can you simplify or clarify ?<br>3) You describe three intervention outcomes in the methods section of your abstract yet report four models in your results. Made it a little confusing .<br>4) your prevalence of sepsis related outcomes in your power calculation were significantly different from your findings. How do you interpret this and how might it effect your findings and power calculation your further validation.work?<br>5) Does missing major trauma data have an impact on the denominator of your tool validation. Ie seriously injured children with reduced parameters but not sepsis. ? If you don't think it would have an effect this should be discussed in the discussion.<br><br>Minor points<br>a) supplement tables should be labelled e-Table one etc

	b) I cannot understand the Natural Language Processing Methods section.<br>c) I think the sample size calculation table should be in the main bulk of the paper.

**REVIEWER NAME**	***Simon Craig***
**REVIEWER AFFILIATION**	None disclosed
**REVIEWER CONFLICT OF INTEREST**	N/A
**AI DISCLOSURE**	No
**DATE REVIEW RETURNED**	10-Mar-2025

**GENERAL COMMENTS**	Thank you for the opportunity to review this interesting paper describing the development and evaluation of machine learning models in children at risk of sepsis.<br>Overall, the paper is clearly written.<br><br>Major comments<br>The title of the paper suggests that the tool is designed to predict “paediatric sepsis”. I am not sure that it does this. Instead, the paper predicts clinician behaviour consistent with treatment of children at risk of sepsis.<br>The outcomes that the authors describe are (1) administration of systemic antibiotics, (2) systemic antibiotics with >20 mL/kg fluid and/or non-elective ventilation, (3) admission for >48 hours of systemic antibiotics. None of these really fit the definition of “sepsis”<br><br>The paper could be improved with a clear definition of “sepsis”. Predicting “outcomes of serious infections and sepsis” is a little unclear. There appears to be some confusion between “serious infection” and “sepsis” and “giving antibiotics”. The outcomes predicted appear to rely on clinician behaviour (i.e. decision to administer antibiotics, decision to administer >20 mL/kg fluids, decision to keep for 2 days), and less on any objective markers of illness in the child.<br><br>Of the three outcomes, outcome one suggests the use of systemic antibiotics. Did this include oral antibiotics? If not, then the term “parenteral” antibiotics may be clearer. Table 1 suggests that “IV” antibiotics were used. Is this correct?<br><br>The definition of “critical care” is a little strange. Administration of 21 mL/kg isn’t necessarily “critical care”, while non-elective mechanical ventilation definitely is. Vasoactive medications are often used in severe sepsis. I do not believe that a decision to give more than 20 mL/kg is enough for a child to be considered “critically ill.”<br>Critical care admission (i.e. ICU admission), or interventions which can only be delivered in a critical care environment (i.e. mechanical ventilation, renal replacement therapy, vasoactive medication) would be a more robust definition of critical care.<br><br>None of the three outcomes (antibiotics, “critical care treatment” or “more than 2 days in hospital”) are consistent with a recognised definition of “sepsis” (organ dysfunction due to infection).<br>Data cleaning section. “Five members of the clinical care team reviewed the data” – does this mean that each one of over 35,000 records were individually reviewed? How was the review

undertaken? Was each one done by a single reviewer? Or was there some double-checking and/or assessment of inter-rater agreement? "The team also examined scanned medical records … for the blood test group." (5,292 records)
What information was available after discharge home? For example, if a patient was discharged home and re-presented with "sepsis" 12-24 hours later resulting in an ICU admission and/or death? Would the first presentation be assessed only against the outcome of the first presentation (e.g. successful discharge home)?

Methods – model evaluation
The study appears to aim to achieve a sensitivity of 72.7%, based on "prior evidence". Please expand on this evidence. Why is a sensitivity of 72.7% acceptable in a high-stakes condition such as sepsis?

Methods – patient and public involvement.
This appears quite generic. Please provide examples of specific input provided by the YPAG. How did they refine the research questions? What input was put into data collection methodologies? What specific ethical considerations in data usage were suggested? How have they participated in dissemination activities of an unpublished paper?

Discussion
The authors note that the Phoenix Sepsis Criteria provide a robust definition of paediatric sepsis, and note that they are not designed for early diagnosis. However, they are useful as a "gold standard" of sepsis diagnosis. The authors appear to consider an "action" (e.g. giving antibiotics, fluids, etc) in response to suspected infection the same as an "outcome"

"The Triage Model, with an AUC of 0.8, has the potential for substantial impact…." How do the authors see this being used? To direct blood tests? Or direct antibiotics?
It may be worth considering determination of which factors at triage predict the taking of blood tests to further risk-stratify children with possible sepsis (rather than predicting the administration of antibiotics).

Minor comments.

Abstract –
Results. Please add some detail on false positive rates across the models. How often did they "overcall" the sepsis intervention?
in the "conclusion" section please add a brief comment as to the applicability of the study findings. Is this ready for widespread use? Or does it need further validation?

Introduction
3rd paragraph: "Our study addresses these challenges…" – change to "Our study aims to address these challenges…" Also, the study does NOT predict "outcomes of serious infections and sepsis" (sepsis is not defined in the study)

	Methods – model training<br>Please provide a reference for SMOTE increasing false positive rates

VERSION 1 – AUTHOR RESPONSE

	Reviewer 1	
1	The abstract should be more explicit about 15 predictive parameters were used for the modelling and these were based on information at triage and information post blood tests.	Now included in the Abstract

2	Some of the information in your intervention section in your abstract (e.g. XGBoost, neural networks, and random forests, Natural Language Processing outputs) was unclear to a reader who does not know this territory - can you simplify or clarify ?	Now simplified in the Abstract
3	3) You describe three intervention outcomes in the methods section of your abstract yet report four models<br>in your results. It made it a little confusing	This has now been clarified and explained more clearly in the Abstract.

4	Your prevalence of sepsis related outcomes in your power calculation were significantly different from your findings. How do you interpret this and how might it affect your findings and power calculation for your further validation work?	The prevalence of sepsis-related outcomes in our power calculation was not substantially different from our findings.<br>Table 1 (Table of Outcomes) presents the prevalence findings from our study (along with their corresponding denominators) that could be used for sample size calculations in the future, validation work:<br>• Model 1: 1138/35,785 (3.2%)<br>• Model 2: 155/4700 (24.2%)<br>• Model 3: 155/4700 (3.3%)<br>• Model 4: 443/4700 (9.4%)<br>In comparison, eTable 3 (Sample size calculation) shows the prevalences used for sample size calculations:<br>• Model 1: 1%<br>• Model 2: 21.6%<br>• Model 3: 3%<br>• Model 4: 9%<br>Importantly, our study exceeded the required sample size for each model.<br>For Model 1, the prevalence used in the power calculation was 1%, derived from a single-centre study conducted in a deprived region of London around 2019. In our study, this prevalence was 3.2%. This difference could potentially be attributed to regional variations and differences in hospital settings — for example, district general hospitals versus tertiary centres, which may see more patients with complex backgrounds, leading to increased IV antibiotic use.<br>As part of Phase 2 of this project, we are conducting prospective data collection across six sites in the UK over an 18-month period. This broader dataset will help address regional variation in prevalence and further strengthen the generalizability of our findings. [Note: Table 3: Model Performance: the prevalences reported relate only to the test set dataset; In case this is confusing to the reader, we could remove that column].

5	Does missing major trauma data have an impact on the denominator of your tool validation. i.e. seriously injured children with reduced parameters but not sepsis. ? If you don't think it would have an effect this should be discussed in the discussion.	We acknowledge that the exclusion of major trauma patients limits the applicability of our sepsis prediction tool to this specific population. However, this decision was intentional, as the models were developed to identify infection-driven deterioration rather than physiological changes associated with major trauma. Including major trauma cases could have introduced confounding variables, given the distinct pathophysiology and clinical management of trauma versus sepsis. Therefore, the prediction models were not designed for use in major trauma patients. We have added this in the Strength and Limitations section of the Discussions.

	Minor Comments	
6	Supplement tables should be labelled e-Table one etc	Tables now labelled as suggested
7	I cannot understand the Natural Language Processing Methods section.	Now simplified in the online Supplement; Natural Language Processing Methods
8	I think the sample size calculation table should be in the main bulk of the paper.	We reached the maximum allowable number of tables and figures in the main manuscript, so the sample size table was placed in the online supplement. We're happy to move this table into the main manuscript, and can shift another table or figure to the supplement upon acceptance for publication, as guided by the Editorial team.
	Reviewer 2	

1	The paper could be improved with a clear definition of “sepsis”. Predicting “outcomes of serious infections and sepsis” is a little unclear. There appears to be some confusion between “serious infection” and “sepsis” and “giving antibiotics”. The outcomes predicted appear to rely on clinician behaviour (i.e.<br>decision to administer antibiotics, decision to administer >20 mL/kg fluids, decision to keep for 2 days), and less on any objective markers of illness in the child	A definition of sepsis and justification of outcome measures now provided under Methods; Outcome Measures. The outcomes being predicted has now been explained more clearly as being aligned with the Improving Pediatric Sepsis Outcomes (IPSO) criteria
2	Of the three outcomes, outcome one suggests the use of systemic antibiotics. Did this include oral antibiotics? If not, then the term “parenteral” antibiotics may be clearer. Table 1 suggests that “IV” antibiotics were used. Is this correct?	Yes, Intravenous antibiotics were considered (including IM/IO). We have corrected and streamlined this within the manuscript throughout where needed.

3	i) The definition of “critical care” is a little strange. Administration of 21 mL/kg isn’t necessarily “critical care”, while nonelective mechanical ventilation definitely is. Vasoactive medications are often used in severe sepsis. I do not believe that a decision to give more than 20 mL/kg is enough for a child to be considered “critically ill.” Critical care admission (i.e. ICU admission), or interventions which can only be delivered in a critical care environment (i.e. mechanical ventilation, renal replacement therapy, vasoactive medication) would be a more robust definition of critical care. ii) None of the three outcomes (antibiotics, “critical care treatment” or “more than 2 days in hospital”) are consistent with a recognised definition of “sepsis” (organ dysfunction due to infection).	i) We understand and acknowledge the complexities surrounding the definition of "critical care." Our use of the term aligns with the Improving Pediatric Sepsis Outcomes (IPSO) criteria, which define critical sepsis based on treatment and interventions rather than a confirmed diagnosis of infection-related organ dysfunction.<br>Specifically, IPSO recognises the administration of IV antibiotic AND three intravenous fluid boluses OR bolus plus vasoactive agent as indicators of critical sepsis.<br>The threshold > 20 mL/kg fluid resuscitation (three or more boluses), while not solely indicative of critical illness, is commonly used in paediatric sepsis protocols as a trigger for closer monitoring and potential escalation to critical care interventions. Moreover, in practice, children receiving >20 mL/kg of intravenous fluids for suspected sepsis are often managed in high-dependency or intensive care settings due to the need for ongoing monitoring and potential further interventions, such as vasoactive support or mechanical ventilation. While we acknowledge that vasoactive medications and renal replacement therapy are markers of severe sepsis, our model aims to capture a broader cohort where early critical care interventions are initiated. This inclusive approach aligns with real-world clinical decision-making, where escalation of care is based on the need for initial resuscitation and stabilisation rather than the presence of confirmed organ dysfunction alone.<br>ii) Justification of Outcome Measures:<br>The outcomes in our model aim to capture varying degrees of sepsis severity, reflecting the spectrum of clinical management seen in paediatric practice.<br>Specifically:<br>Antibiotic model: This outcome aligns with the IPSO suspected infection where the administration of antibiotics, blood tests, and microbiology investigations reflect a clinical concern for sepsis. Critical care model: aligns with the IPSO critical sepsis criteria<br>Serious infection model (admission > 2 days): reflect children considered by paediatricians to likely have serious or invasive bacterial infections or have positive bacteriology.<br>Our approach aims to identify children who received treatment consistent with varying severities of sepsis, recognising that sepsis exists along a continuum. While these outcomes may not map perfectly onto traditional sepsis definitions (e.g Phoenix Criteria), they reflect pragmatic clinical decisions and management pathways for children with suspected sepsis.

4	Data cleaning section. “Five members of the clinical care team reviewed the data” – does this mean that each one of over 35,000 records were individually reviewed? How was the review undertaken? Was each one done by a single reviewer? Or was there some doublechecking and/or assessment of inter-rater agreement? “The team also examined scanned medical records … for the blood test group.” (5,292 records	Given the scale of the dataset and logistical constraints, it was not feasible to review over 35,000 individual records in full. Instead, we implemented a structured approach to ensure efficient data management.<br>For the main dataset of over 35,000 patients, the data was divided into five sections, with each section assigned to one of the five clinical team members, who had secure access only to their allocated portion. The reviewers filtered the dataset in Excel using criteria for example: age (>16 years), discharge diagnosis, and patients not triaged or who left before being seen to identify ineligible cases. These ineligible cases were then flagged and subsequently removed by the data scientist.<br>A separate dataset was created for the subgroup of patients who had undergone blood tests (n = 5,292). This dataset was similarly divided into five sections, with each reviewer having secure access to their allocated records. For these patients, scanned medical records were examined to extract the clinical information:<br>• Duration of intravenous antibiotic administration<br>• Receipt and volume of fluid boluses<br>• Ventilation details<br>• Paediatric Intensive Care Unit (PICU) admissions<br>• In-hospital deaths<br>• Any missing vital signs or blood test results Due to time and resource constraints, doublechecking of records and formal assessment of interrater agreement were not conducted. However, any uncertainties or discrepancies were discussed and resolved with the support of Johanna Bellamy (Senior Nurse, acknowledged in the manuscript).
5	What information was available after discharge home? For example, if a patient was discharged home and represented with “sepsis” 12-24 hours later resulting in an ICU admission and/or death? Would the first presentation be assessed only against the outcome of the first presentation (e.g. successful discharge home)?	During the model development, each attendance was considered as a new episode or new encounter. We had flags within each episode of re-attendance within 24 hours and re-attendance within 48 hours. None of PICU admissions (n=45) or deaths (n=3) had attendance within 48 hours.

6	Methods – model evaluation: The study appears to aim to achieve a sensitivity of 72.7%, based on “prior evidence”. Please expand on this evidence. Why is a sensitivity of 72.7% acceptable in a high-stakes condition such as sepsis?	This has now been expanded and explained in the section Methods – model evaluation. As these are prototype models, we are going to explore the best sensitivity/ specificity combinations during the external validation/ development of the models,using multi-centre big data in Phase 2 (ongoing).
7	Methods – patient and public involvement. This appears quite generic. Please provide examples of specific input provided by the YPAG. How did they refine the research questions? What input was put into data collection methodologies? What specific ethical considerations in data usage were suggested? How have they participated in dissemination activities of an unpublished paper?	We have addressed your queries and provided a more detailed description in the NEW Patient and Public Involvement (PPI) section of the online Supplement.

8	Discussion - The authors note that the Phoenix Sepsis Criteria provide a robust definition of paediatric sepsis, and note that they are not designed for early diagnosis. However, they are useful as a "gold standard" of sepsis diagnosis. The authors appear to consider an "action" (e.g. giving antibiotics, fluids, etc) in response to suspected infection the same as an "outcome"	We recognize that the Phoenix Sepsis Criteria (PSC) were developed to provide a robust definition of paediatric sepsis, with a particular focus on identifying life-threatening organ dysfunction in children with suspected or confirmed infection. As highlighted by Jabornisky et al. (2024), the PSC was not designed as a screening tool or an early warning system, nor should it be misconstrued as a sepsis test. Crucially, the score does not predict which children are at risk of developing sepsis. Identifying the precise moment when the immune system begins to deregulate — and determining whether clinicians should wait for organ dysfunction to diagnose sepsis — remains a significant challenge.<br>Our models aim to predict an event before it occurs, using information gathered in the preceding stages. In the absence of a singular biomarker to pinpoint the onset of sepsis, clinical interventions such as sepsis screening, antibiotic administration, or fluid bolus delivery are being used as proxies for early sepsis recognition. This approach aligns with the reality of clinical practice, where these actions often reflect clinician suspicion.<br>Recent literature emphasizes that machine learning models should be trained on outcome labels grounded in real-world clinical judgment rather than relying exclusively on formal sepsis definitions, whose early diagnostic utility is uncertain (Lindner et al., 2023). We aim to leverage clinician-driven actions as indicators to reflect the complex decision-making involved in early sepsis recognition, offering a more practical basis for developing predictive models.

9	“The Triage Model, with an AUC of 0.8, has the potential for substantial impact….” How do the authors see this being used? To direct blood tests? Or direct antibiotics? It may be worth considering the determination of which factors at triage predict the taking of blood tests to further riskstratify children with possible sepsis (rather than predicting the administration of antibiotics).	The Triage Model predicts Outcome 1 — the delivery of the paediatric sepsis care bundle (blood tests, microbiology, and antibiotics) — using information available at triage. The model is not intended to mandate antibiotics but rather to support early identification of children at higher risk of sepsis, prompting timely clinician review and consideration of further investigation.<br>We recognise that children can show significant physiological responses to benign conditions like fever, anxiety, or pain. Early measures, such as antipyretics or a calming environment, often improve vital signs, potentially reducing risk scores later. Therefore, the Triage Model serves as an initial screening tool to identify children who may benefit from early clinical escalation/ huddle after triage.<br><br>Additionally, Phase 2 of this project is collecting serial observations throughout the ED visit to develop realtime risk prediction tools, providing a more dynamic risk assessment beyond triage.
	Minor comments.	
10	Abstract – Results. Please add some detail on false positive rates across the models. How often did they “overcall” the sepsis intervention?	We appreciate the reviewer’s request to include false positive rates in the Abstract. However, due to the word count limitations, adding this detail would require additional context to avoid misinterpretation — context that is already provided in the Results and<br>Discussion sections. Including these details in the Abstract could make it overly dense and detract from highlighting the key findings to the reader. However, if absolutely needed by the Editorial team, we would be happy to revise and add this to the Abstract.
11	In the “conclusion” section please add a brief comment as to the applicability of the study findings. Is this ready for widespread use? Or does it need further validation?	Now added to the conclusion section of the Abstract.

12	Introduction - 3rd paragraph: "Our study addresses these challenges…" – change to "Our study aims to address these challenges…" Also, the study does NOT predict "outcomes of serious infections and sepsis" (sepsis is not defined in the study)	Changes made as recommended. Sepsis is now defined in the Methods section.

13	Methods – model training: Please provide a reference for SMOTE increasing false positive rates:	Reference was added. Alkhawaldeh IM et al. doi: 10.5662/wjm.v13.i5.373.

VERSION 2 - REVIEW

**REVIEWER NAME**	***Colin Powell***
**REVIEWER AFFILIATION**	None disclosed
**REVIEWER CONFLICT OF INTEREST**	N/A
**AI DISCLOSURE**	No
**DATE REVIEW RETURNED**	16-Apr-2025

**GENERAL COMMENTS**	Thank you<br>You have clarified your sample size prevalences further.<br>The authors have addressed my comments thoroughly<br>I think the sample size table, e table 3 in the supplement should be in the main paper. This will put the number of tables over the limit, but I think it helps the reader understand the paper as it is read.<br>Thanks nothing more to add

**REVIEWER NAME**	***Simon Craig***
**REVIEWER AFFILIATION**	None disclosed
**REVIEWER CONFLICT OF INTEREST**	N/A
**AI DISCLOSURE**	No
**DATE REVIEW RETURNED**	06-Apr-2025

**GENERAL COMMENTS**	Thank you for the opportunity to review this revised paper, and for the effort you have made to address the initial feedback provided.<br><br>Thank you for more explicitly defining "sepsis" (which the authors have essentially defined as taking blood tests, giving fluid boluses + antibiotics), rather than "sepsis" according to definitions of organ dysfunction.<br>It may be better to make this explicit in the title – the study is about predicting sepsis treatment, rather than predicting sepsis. Perhaps "Designing a Tool to Predict Paediatric Sepsis Treatment using Machine Learning"<br><br>One minor comment

	Table 1 – please add percentage to each column (i.e. number and percentage)

VERSION 2 – AUTHOR RESPONSE

Reviewer 1 (Dr Powell):

We have included the Sample size table (as Table 1) from the supplementary materials into the main manuscript as recommended. We agree that its inclusion enhances the clarity of the paper and better supports the readers' understanding.

Reviewer 2 (Dr Craig):

In response to the suggestion regarding the title, we have updated it to: “Predicting Sepsis Treatment Decisions in the Paediatric Emergency Department Using Machine Learning: The AiSEPTRON Study”.

Regarding the suggestion to add percentages to Table 2 (previously Table 1); we have provided a detailed response below for your reference:

The authors had considered adding percentages during the development of the manuscript. However, Table 2 includes a nested structure in which:

Column 2 (Patients triaged, n=35,795) is subset of the overall attendances including injuries (n=46,553).

Column 3 (Patients that had blood tests, n=4,700) is a subset of Column 2.

Columns 4–6 are subsets of Column 3, with each reflecting different clinical outcomes (IV antibiotics, critical care, or longer length of stay).

Because of this, adding percentages to each column could be confusing without detailed clarification of denominators (e.g. whether percentages are based on total attendance, total triaged, those tested, or specific outcomes). We were concerned that this might compromise the clarity of the table.

We are happy to revise the table to include percentages if preferred. For example, we can: add percentages with explicit footnotes in the legend clarifying the denominator used for each column.

To maintain clarity, we chose to present raw counts and describe the hierarchical relationships in the text adjacent to the table. We believe that adding percentages may not be necessary and would welcome your guidance on which option would be most appropriate for the readers.

VERSION 3 - REVIEW

**REVIEWER NAME**	***Colin Powell***
**REVIEWER AFFILIATION**	None disclosed
**REVIEWER CONFLICT OF INTEREST**	N/A
**AI DISCLOSURE**	No
**DATE REVIEW RETURNED**	23-Apr-2025

**GENERAL COMMENTS**	Thankyou<br>No further comments
